# Food frequency questionnaires developed and validated in Brazil: A scoping review protocol

**DOI:** 10.1371/journal.pone.0294450

**Published:** 2023-11-20

**Authors:** Acsa Nara de Araújo Brito Barros, Maria Luisa do Nascimento Felipe, Lucia Leite-Lais, Lucia Fátima Campos Pedrosa

**Affiliations:** 1 Graduate Program in Health Sciences, Federal University of Rio Grande do Norte, Natal, Brazil; 2 Graduate Program in Nutrition, Federal University of Rio Grande do Norte, Natal, Brazil; 3 Department of Nutrition, Federal University of Rio Grande do Norte, Natal, Brazil; Fiocruz Mato Grosso do Sul, BRAZIL

## Abstract

A food frequency questionnaire (FFQ) is used to assess habitual food and nutrient intake. The choice of a FFQ should consider the study objectives, instrument particularities, target population, and geographic region. Over the past few years, FFQs have been constructed and validated in Brazil for children, adolescents, adults, athletes, and individuals with specific clinical conditions. The aim of this scoping review is to map the food frequency questionnaires developed and validated in Brazil. The Population-Concept-Context (PCC) framework was used for search strategy and defined as P—not applicable (open), C—food frequency questionnaire, and C—Brazil. FFQ validation studies performed with healthy or sick people will be included, regardless of clinical condition, age, sex, or region in the country. Studies with populations from other countries will be excluded. The review will be conducted in accordance with JBI (formerly known as Joanna Briggs Institute) methodology for scoping reviews. Search databases will include PubMed/MEDLINE, LILACS, Embase, Web of Science (ISI), Scopus, and Google Scholar. Data extraction will be performed by two independent reviewers and discrepancies resolved by a third reviewer. In order to improve the understanding and contextualization of the studies, a description of the results and presentation in tables and figures will be provided. Applications and implications for future research, practices, and policies will be discussed. Our protocol is registered through the Open Science Framework (doi 10.17605/OSF.IO/G5J3K).

## Introduction

The human diet is considerably complex and it has been studied in nutritional epidemiology due to the possible influence of dietary aspects on the occurrence of chronic non-communicable diseases [1, 2].

Food consumption can be assessed in several ways, including intake of chemical compounds, foods, food groups, and dietary patterns [3]. Dietary assessment methods include retrospective and prospective methods. All methods have strengths and limitations, so the choice of the most appropriate method will depend on the objective of the evaluator. Among the prospective methods, the most used is the food record. Among the retrospective methods, the 24-hour dietary recall (24h recall) and the food frequency questionnaire (FFQ) are commonly used. The 24h recall assesses short-term diet and the FFQ assesses long-term diet [4].

The assessment of long-term food intake has been widely used under the assumption that the estimation of the usual diet, practiced over weeks, months or years, constitutes a more important exposure factor than the estimation of the actual food intake [5]. In this context, despite the new perspectives of innovative methods of assessing food consumption [2], the FFQ is still used in epidemiological studies to assess the intake of chemical compounds (nutrients, non-nutritional compounds, and others), specific foods or food groups, with low cost and easy administration [6]. The FFQ is composed of a fixed list of foods in which the respondent indicates the consumption of a certain portion of those foods during a certain period of time. As this method depends on the interviewee’s memory, imprecise answers or possible omissions are some disadvantages of the FFQ [5, 7].

The choice of a FFQ should be made carefully and it must consider the objectives and the particularities of the instrument such as type, scope of dietary assessment (global or specific), and administration approach. Furthermore, as the human diet varies according to geographic region, age, and cultural aspects, FFQs need to be specific and validated for the target population [8]. However, depending on the researcher’s objective or goals in clinical practice, elaborating a FFQ can be expensive and unnecessary work, since it is possible to adapt existing questionnaires, followed by a validation for the population studied. However, the instrument must be minimally adequate to the population under evaluation [5].

In a country with continental dimensions like Brazil (8,516,000 km^2^), divided into five regions, the food particularities of each region present considerable variations derived from cultural roots arising from colonization. A non-systematic review developed by Pedraza and Menezes (2015) [9] was published many years ago with the objective of identifying the studies that developed and/or validated FFQ in Brazil until 2013. The authors discussed the FQQ validation methods and its main results, analyzing the most appropriate instruments and verifying the usefulness of such FFQs in the country.

In recent years, FFQs have been constructed and validated in Brazil for specific populations such as children [10], adolescents [10–12], adults [12, 13], athletes [14] and individuals with specific clinical conditions [15, 16]. Considering the diversity of FFQs constructed, we observed the need to map the FFQs that exist in Brazil to date, identifying the objectives, particularities, and the population for which they are intended. This mapping will help Brazilian researchers and clinicians in consulting the existence of specific FFQs according to their needs. A preliminary search of PubMed/MEDLINE, Cochrane Database of Systematic Reviews, and JBI Evidence Synthesis was performed and no current or ongoing systematic or scoping reviews on the topic were identified. Thus, this scoping review intends to update the identification of published FFQs, which were developed and validated in Brazil, using a systematic methodology capable of answering a comprehensive research question.

## Materials and methods

The proposed scoping review will be conducted according to the approach proposed by the JBI (formerly known as Joanna Briggs Institute) for scoping reviews [17] and the PRISMA extension for Scoping Reviews (PRISMA-ScR) (S1 Checklist) [18]. Our protocol is registered through the Open Science Framework (doi 10.17605/OSF.IO/G5J3K).

### Research question

This scoping review seeks to answer the following question: Which FFQs have been developed and validated in Brazil?

### Eligibility criteria

To construct the research question, the Population-Concept-Context (PCC) framework was used for search strategy and defined as P—not applicable (open), C—food frequency questionnaire, and C—Brazil (Table 1).

**Table 1:** PCC framework used in the scoping review.

PCC	Description
Population	Open
Concept	Food frequency questionnaires that assess the intake of energy, nutrients (specific or not), isolated foods or food groups
Context	Brazilian population, regardless of health status, clinical condition, age, sex or region in the country

Considering the nature of the research question, the population of the PCC framework was not applicable (open). Other types of dietary assessment methods or studies with populations from other countries will be excluded.

### Types of sources

This scoping review will consider FFQ development and validation studies. Other study designs to evaluate food consumption are not aligned with the purpose of this review.

### Search strategy

The search strategy will aim to locate both published and unpublished studies. Studies performed up to February 2023 will be included. Considering the comprehensive nature of this review, no restrictions of date or language will be imposed in the search strategy.

An initial search in PubMed/MEDLINE, Embase, LILACS, Web of Science (ISI), Scopus, and Google Scholar was performed to identify some reports on the topic. Key words and indexing terms of these reports were used to develop a search strategy for PubMed/MEDLINE, Embase, LILACS and Google Scholar Databases (see example in Table 2). The search equations will be adapted for each database and/or information source included.

**Table 2:** Search strategy for PubMed/MEDLINE database.

Date		Data base	Search results
10/23/2023	(((("Surveys and Questionnaires"[Mesh] OR (Questionnaires and Surveys) OR (Survey*) OR (Questionnaire Design*)) OR ("Diet Records"[Mesh] OR (Diet* Record))) AND ((FFQ) OR (food frequency questionnaire*))) AND ("Reproducibility of Results"[Mesh] OR (Validity Result*) OR (Validity and Reliability))) AND ("Brazil"[Mesh])	PubMed	113

Sources of unpublished studies to be searched include national theses and dissertation databases. In addition, manual searches of the reference lists of the included articles will be performed to identify additional studies that were not retrieved through the search equations.

### Study selection

After the search, all identified reports will be entered into the Rayyan online application (Qatar, 2022) and duplicates will be removed. Titles and abstracts will be selected by two independent reviewers for evaluation according to the inclusion criteria. Potentially eligible articles will be retrieved in full. The full-text articles will be evaluated in detail by the reviewers, according to the inclusion criteria. The reasons for excluding full-text articles that do not meet the inclusion criteria will be recorded and reported. At each step, any disagreements that arise between reviewers will be resolved by a third reviewer. A narrative description of the search and study inclusion process accompanied by a flowchart of the review process (from the PRISMA-ScR statement) will be provided [17].

### Data extraction

Two independent reviewers will perform data extraction using a form developed by the authors according to the model (Table 3 and S1 File). The extracted data will include details about the concept, context, study methods, and key findings relevant to the review question.

**Table 3:** Description of variables for data extraction.

Variables	Description
Author and year	Authors and year of publication
Brazil region	State and region of the country in which the instrument was validated
Study population	Characteristics of the target population: age, sex, and clinical condition or status
**FFQ features**	
Type	Quantitative, semi-quantitative, qualitative
Foods and/or nutrients assessed	Specific foods, food groups, and/or nutrients assessed
Number of items	Number of instrument questions
Food portions and frequency	Size of portions consumed and frequency category such as day, month, year
Visual support	Presence of food photographs to help to describe portion sizes
Method of administration	Self-administered or administered by an interviewer
Validation method	Method used for validation

The preliminary data extraction form will be modified and revised as necessary for application in the scoping review. Any disagreements that arise between reviewers will be resolved by a third reviewer. If necessary, article authors will be contacted to request missing or additional data.

### Data analysis and presentation

Based on the extracted results, a description of the characteristics of the studies, the population, and the FFQs developed and validated will be performed, as well as a categorized presentation of the types of FFQ found, according to the target population. The results will be reported using resources such as tables and figures to improve the understanding and contextualization of the results. Also, applications and implications for future research, practices and policies will be discussed.

### Ethics and dissemination

Ethical approval is not required as no human research participants will be involved in this review. Review findings will be published in a peer-reviewed journal, and presented at a number of scientific conferences.

## Discussion

This review will use a systematic search strategy and the JBI standardized methodology for scoping reviews. In addition, it will consider articles from a broad range of settings, interventions, and study designs that will lead to insights for a wide array of healthcare research audiences.

It will be beneficial to map the existing FFQs in Brazil, a huge and culturally diverse country. This review will contribute to the design of research on food consumption, facilitating the identification of existing FFQs in the area of interest for the study, encouraging production or adaptation to specific populations, foods/food groups, and nutrients, or reducing unnecessary FFQ development. Furthermore, the results of this review will guide the use of specific FFQs in the individual or group clinical assessment of habitual food consumption in Brazil.

The limitations of this study are related to the comprehensive and exploratory nature of the scoping review. In case of necessary changes to this protocol after publication, the date, nature, and justification of such changes will be provided.

## Supporting information

S1 Checklist PRISMA-ScR—Preferred Reporting Items for Systematic reviews and Meta-Analyses extension for Scoping Reviews checklist. (DOCX)

S1 File Data extraction tool. (DOCX)
