# Supplementary material for: Food frequency questionnaires developed and validated in Brazil: A scoping review protocol
Source: PLoS One. 2023 Nov 20;18(11):e0294450. doi: 10.1371/journal.pone.0294450 (PMC10659153; doi:10.1371/journal.pone.0294450)
Supplement: S1 File — (DOCX) [file pone.0294450.s002.docx]

***Food frequency questionnaires developed and validated in Brazil: a scoping review protocol***

Barros et al. (2023)

**DATA EXTRACTION FORM**

1. **Data extractor's name**
2. **Extraction date**
3. **Article title**
4. **Author**
5. **Publication year**
6. **DOI/Reference**
7. **Journal**
8. **Brazilian region of study**
9. **Study type**
   1. Food frequency questionnaire (FFQ) development, FFQ validation
10. **Study objective**
11. **Population**
    1. Gender
    2. Age/Age Group
    3. Health condition
12. **Characteristics of the FFQ**
    1. Type: quantitative, semiquantitative, qualitative
    2. FQQ objective
    3. Number of itens: number of instrument questions
    4. Food portions and frequency: size of portions consumed and frequency category such as day, month, year
    5. Visual support: presence of food photographs to help to describe portion sizes
    6. Method of administration: Self-administered or administered by an interviewer
    7. Validation method:method used for validation
13. **Observations.**
